# “Investigation of the Effect of Sleep Hygiene Training on Obstructive Sleep Apnea Symptoms, Fatigue, and Sleep Quality In Patients With Atrial Fibrillation”

**DOI:** 10.1002/clc.70128

**Published:** 2025-03-31

**Authors:** Besey Ören, Busra Zehra Buyukkilic, Semiha Akin Eroglu, Ahmet Lutfullah Orhan

**Affiliations:** ^1^ Department of Internal Medicine Nursing University of Health Sciences, Hamidiye Faculty of Nursing Istanbul Turkey; ^2^ Department of Cardiology University of Health Sciences, Hamidiye Medical School Istanbul Turkey

**Keywords:** Atrial Fibrillation, Education, Fatigue, Obstructive Sleep Apnea, Sleep Hygiene

## Abstract

**Objective:**

Sleep hygiene education is widely used as a coping strategy for sleep disorders and is known to be an effective, and side‐effect‐free approach. To improve sleep quality by reducing the symptoms and fatigue level experienced by patients with OSAS with sleep hygiene education.

**Method:**

The study was a single‐center, randomized controlled research. A structured training program was applied face‐to‐face to the intervention group. Received inpatient treatment for atrial fibrillation in the Cardiology Service of a Training and Research Hospital between June 2023 and December 2023.

**Results:**

No significant difference was found between the MOS Sleep Scale scores of the control group and the intervention group at 1 month in terms of sleep disturbance, sleep adequacy, shortness of breath, sleepiness, snoring, and sleep duration subscales. Fatigue, concentration, motivation, and physical activity scores of the intervention group patients were lower at the end of the 1st month.

**Conclusion:**

Sleep hygiene education has a positive effect on sleep duration in patients with Atrial Fibrillation, however it is ineffective in ensuring high quality sleep.

## 1 Introduction

Atrial fibrillation (AF) is the most common type of arrhythmia among cardiac rhythm disorders [1]. AF is known to be associated with structural heart disease, hypertension, diabetes, and obesity, as well as extra‐cardiac factors such as obstructive sleep apnea syndrome [2]. The prevalence of OSAS in patients diagnosed with AF is unknown because OSAS is very difficult to diagnose [3]. In the sleep heart health study, the prevalence of AF was four times higher in patients with an apnea‐hypopnea index (AHI) of 30/h or more compared to patients without sleep problems, independent of other risk factors [4].

When Obstructive Sleep Apnea Syndrome is not treated, the recurrence rate has been reported to be high in AF patients who underwent cardioversion or catheter ablation. In patients receiving effective OSAS treatment, the risk of AF recurrence after cardioversion was found to decrease by 50% [5]. The study data of Fein et al. in 2013 showed that the need for antiarrhythmic drugs used in AF treatment decreased after OSAS treatment [6]. Sleep hygiene practices are effective methods in the prevention of OSAS‐related problems. Sleep hygiene education is widely used in strategies to cope with sleep disorders and is an effective, inexpensive, and side‐effect‐free approach to the management of sleep disorders [7, 8]. Although factors related to sleep hygiene affect the symptoms experienced by patients with OSAS, there are very few studies investigating the relationship between sleep hygiene and OSAS symptoms [9]. Wang et al. stated that irregular sleep duration and poor sleep quality significantly increased the risk of death due to a cardiovascular event in a study conducted in 2020 [10]. Fank et al. (2024) suggested in their study that structured sleep hygiene training would effectively reduce OSAS symptoms [11].

Sleep hygiene training could improve sleep quality by reducing the symptoms and fatigue levels experienced by patients with OSAS. The results of the study are important to draw attention to the deficiency in the literature on these patients. In addition, the results of this study contribute to the care that nurses can apply to ensure the sleep hygiene of OSAS patients.

## 2 Methods

The study was a single‐center, randomized controlled, interventional research.

Structured face‐to‐face sleep hygiene education given to patients with atrial fibrillation and OSAS symptoms affects sleep quality.

Structured face‐to‐face sleep hygiene education given to patients with atrial fibrillation and OSAS symptoms affects fatigue.

Structured face‐to‐face sleep hygiene education given to patients with atrial fibrillation and obstructive sleep apnea symptoms affects OSAS symptoms.

The study was conducted in the cardiology service of a training and research hospital.

The study population consisted of all individuals who were hospitalized in the cardiology service of a training and research hospital. The study sample consisted of a total of 62 patients, 30 intervention (experimental) and 32 control groups, randomly selected from among the patients who met the inclusion criteria and received inpatient treatment for atrial fibrillation in the Cardiology Service of a Training and Research Hospital between June 2023 and December 2023.

*Inclusion criteria for the research sample:*

- Diagnosed with atrial fibrillation,
- Symptoms of Obstructive Sleep Apnea Syndrome (such as snoring, stopping breathing during sleep, sleepiness during the day),
- Speaks and understands Turkish,
- Who volunteered to participate in the research,
- Patients aged between 18 and 75 years were included.

Individuals diagnosed with any psychiatric illness were not included in the study sample.

For this study, the effect size was calculated based on 0.8 (large effect size). Power analysis was performed using the G*Power (v3.1.9.7) program to determine the sample size. For intergroup evaluations, when the effect size was considered large (*d* = 0.8), *α* = 0.05 and power was 90%, it was determined that there should be at least 28 cases in each group. Since it was thought that there might be case losses during the study, it was decided to recruit 32 people for both groups. Two people from the intervention group dropped out at the beginning of the study.

The study sample consisted of 30 intervention (experimental) and 32 control group patients selected according to their protocol numbers. While selecting the sample, the odd numbers constituted the intervention group, and the even numbers constituted the control group according to the protocol numbers.

### 2.1 Patient Information Form

This form, which includes questions to determine the individual's age, gender, height, weight, neck circumference, educational status, economic status, marital status, presence of chronic diseases, and medications used continuously, smoking habits and sleep quality.

### 2.2 Berlin Questionnaire

It is a questionnaire created with a consensus reached at the Sleep in Primary Care Conference held in Berlin, Germany in 1996. The validity and reliability study of the Berlin Form for our country was conducted by Acar et al. in 2013. In the study investigating the sensitivity of the “Berlin Form” for use as a screening test for Obstructive Sleep Apnea Syndrome (OSAS), the sensitivity was found to be 87.9% [12]. The Berlin Form includes a total of 10 questions under three categories. The first category includes snoring and apnea (5 items), the second category includes daytime sleepiness (4 items) and the third category includes blood pressure/obesity (1 item). In the evaluation of the form, if two or three categories are positive, it indicates that the patient is at high risk for OSAS, and if only one category is positive, it indicates that the patient is at low risk for OSAS [13].

### 2.3 MOS Sleep Scale (Medical Outcomes Study Sleep Scale [MOS])

The MOS Sleep Scale was developed by Hays et al. in 2005 to assess sleep disturbance in chronic diseases. In a Turkish validity and reliability study conducted by Akçay et al. in 2019 with 120 patients with OSAS and 90 healthy individuals, the Cronbach α internal consistency coefficient of the MOS Sleep Scale was found to be 0.82 [14, 15].

### 2.4 Fatigue Scale (Checklist Individual Strength Questionnaire [CIS‐T])

Developed by Vercoulen et al. in 1999, it is the most widely used questionnaire worldwide to assess chronic fatigue. As a result of the reliability analysis of the Turkish version of the CIS‐T questionnaire conducted by Ergin in 2009, Cronbach's alpha coefficient *α* = 0.87 was found [12, 16]. According to this scale, fatigue is assessed in four sub‐dimensions: subjective perception of fatigue decreased concentration, decreased motivation, and decreased physical activity. The questionnaire consists of 20 statements measuring the individual's fatigue in the last 2 weeks. A 7‐point scale is used for the answers [16].

### 2.5 Sleep Hygiene Index (SHI)

The Sleep Hygiene Index was developed by Mastin et al. in 2006. Its Turkish validity and reliability were performed by Güzel Özdemir et al. in 2015. Cronbach's alpha internal consistency coefficient was calculated as 0.70 [17]. The questionnaire consists of 13 questions. The questions are in the form of a 5‐point Likert scale. The scale score varies between 13 and 65 and higher scores indicate that participants have worse sleep hygiene [18].

**Figure 1:**
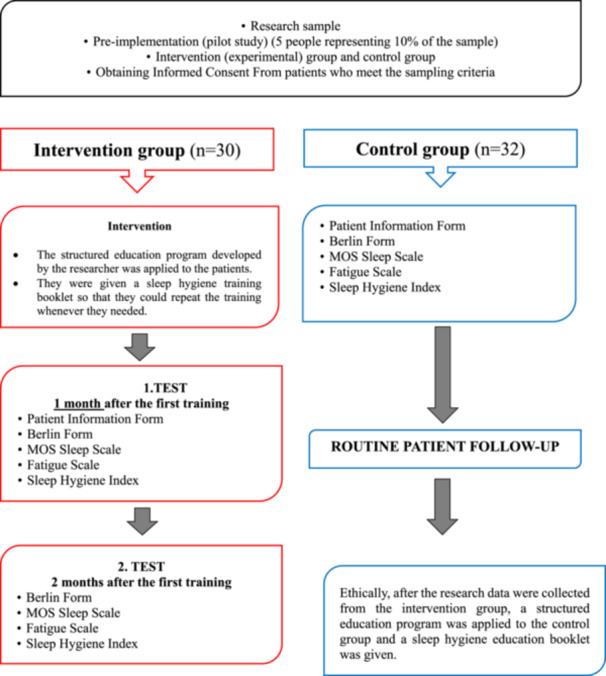
Research Process Flow. The interventions and evaluations are summarized in Figure 1.

The training and training booklet was prepared by the researchers according to the needs of the patients in line with current research and evidence‐based treatment guidelines. A pilot study was conducted on 5 patients who met the inclusion criteria for the scales to be used and the structured education program. The data in the pre‐application were not included in the study. The interventions and evaluations to be applied to the Intervention and Control group within the scope of the protocol are summarized in Figure 1.

Approval of the Clinical Research Ethics Committee (dated 25.05.2023 and numbered 11/18) and permission from the institution where the study was conducted were obtained. Informed Voluntary Consent Form was obtained verbally and in writing from each individual.

Data were analyzed with the SPSS 25.0 (Statistical Program for Social Sciences) package program. The compatibility of continuous data with normal distribution was evaluated using the Shapiro‐Wilk test. Parametric tests were used for data conforming to normal distribution and nonparametric tests were used for data not conforming to normal distribution.

The relationships between the data were evaluated by Spearman or Pearson correlation tests, and the effects of independent variables on the dependent variable were evaluated by regression analysis. Wilcoxon Signed Rank Test was used to evaluate the difference between the scores of the intervention (experimental) and control groups on the scales and their sub‐dimensions in the pre and post‐tests. The statistical significance level was accepted as *p* < 0.05.

## 3 Results

There was no statistically significant difference between the intervention group and the control group in terms of sociodemographic characteristics (*p* > 0.05) (Table 1).

**Table 1:** Comparison of sociodemographic characteristics of the intervention and control groups.

		Intervention group (n = 30)		Control group (n = 32)		
		n	%	n	%	*p*
Gender	Woman	13	43.3	14	43.8	0.974
	Male	17	56.7	18	56.3	
Marital status	Married	30	100	31	96.9	1.000
	Single	0	0	1	3.1	
Education	Literate/Primary School	7	23.3	8	25	0.970
	Secondary Education	11	36.7	12	37.5	
	High School	9	30	8	25	
	Associate degree/undergraduate	3	10	4	12.5	
	Master's degree and above	0	0	0	0	
Employment status	Working	7	23.3	11	34.4	0.338
	Not working	23	76.7	21	65.6	
Smoking	Yes	15	50	16	50	1.000
	No	15	50	16	50	
Chronic disease	Yes	24	80	28	87.5	0.502
	No	6	20	4	12.5	
Medication use	Yes	22	73.3	23	71.9	0.898
	No	8	26.7	9	28.1	
History of stroke	Yes	4	13.3	3	9.4	0.703
	No	26	86.7	29	90.6	
Sleep status	Very good	0	0	0	0	0.560
	Good	11	36.7	10	31.3	
	Middle	13	43.3	13	40.6	
	Bad	6	20	7	21.9	
	Very Bad	0	0	2	6.3	
		Mean ± SD		Mean ± SD		
Age	Mean ± SD	60.0 ± 8.1		60.5 ± 8.1		0.882
	Min‐max	(44–74)		(44–74)		
Body mass index	Mean ± SD	29.0 ± 3.7		27.5 ± 3.1		0.023
	Min‐max	(20.5–34.6)		(22.5–27.5)		
Neck circumference	Mean ± SD	36.5 ± 3.9		36.5 ± 3.7		0.983
	Min‐max	(27–44)		(29–43)		
Daily sleep duration (Hours)	Mean ± SD	5.4 ± 0.9		5.2 ± 1.4		0.396
	Min‐max	(3–8)		(3–9)		

Pearson Chi‐Square Test *p* < 0.05.

Mann Whitney‐U Test *p* < 0.05.

There was no statistically significant relationship between the age, neck circumference, and sleep duration of the patients in the intervention group and the control group (*p* > 0.05).

### 3.1 Comparison of the Intervention and Control Group Scores Following the Structured Education Program

The comparison of the control group and the intervention group (Table 2).

**Table 2:** Comparison of the control group and intervention group according to the scores obtained from the scales.

		Control group	Intervention group			
			After training 1st month	*p*	After training 2nd month	*p*
		n (%)	n (%)		n (%)	
Berlin Form	Low	9 (28.1)	11 (36.7)	0.472	12	0.47
	High	23 (71.9)	19 (63.3)		18	
MOS Sleep Scale Subscales		Control group	1st month after training	*p*	2nd month after training	*p*
*UPI1*	Mean ± SD	3.69 ± 0.66	3.87 ± 0.54	0.285	3.92 ± 0.55	0.18
	Median [IQR (25–75)]	4 (3.5–4.125)	4 (3.5–4.20)		4 (3.5–4.33)	
*UPI2*	Mean ± SD	3.49 ± 0.52	3.68 ± 0.45	0.183	3.72 ± 0.43	0.12
	Median [IQR (25–75)]	3 (3.3–3.88)	4 (3.33–4)		4 (3.36–4)	
*Sleep disorder*	Mean ± SD	51.37 ± 10.42	57.17 ± 10.4	0.029	59.03 ± 9.81	0.005
	Median [IQR (25–75)]	50 (42–58)	58 (49–64)		60 (53–67)	
*Sleep adequacy*	Mean ± SD	62.47 ± 15.45	53.57 ± 15.9	0.044	49.17 ± 13.96	0.001
	Median [IQR (25–75)]	58 (52–67)	58 (42–67)		50 (39.75–58)	
*Shortness of breath*	Mean ± SD	70.91 ± 18.82	83.33 ± 17.46	0.004	83.9 ± 17.73	0.003
	Median [IQR (25–75)]	67 (54.25–83)	83 (67–100)		83 (67–100)	
*Sleep state*	Mean ± SD	67.22 ± 14.9	74.8 ± 14.42	0.059	77.4 ± 13.56	0.007
	Median [IQR (25–75)]	67 (56–83)	78 (61–84.5)		78 (67–89)	
*Snoring*	Mean ± SD	35.91 ± 24.25	38.8 ± 22.86	0.323	38.8 ± 22.86	0.32
	Median [IQR (25–75)]	33 (17–33)	33 (29–37.25)		33 (29–37.25)	
*Sleep duration*	Mean ± SD	4.25 ± 1.13	5.00 ± 1.05	0.217	5.03 ± 1.06	0.09
	Median [IQR (25–75)]	4 (3.25–5)	5 (4–6)		5 (4–6)	
Fatigue Scale Subscales		Control group	1st month after training	*p*	2nd month after training	*p*
*Fatigue*	Mean ± SD	4.55 ± 1.03	4.01 ± 1.02	0.062	3.75 ± 0.9	0.003
	Median [IQR (25–75)]	4 (3.90–4.93)	4 (3.12–4.75)		4 (3.09–4.25)	
*Concentration*	Mean ± SD	4.24 ± 0.75	3.89 ± 0.8	0.050	3.67 ± 0.75	0.003
	Median [IQR (25–75)]	4 (3.85–4.75)	4 (3.35–4.25)		4 (3.2–4.2)	
*Motivation*	Mean ± SD	3.9 ± 0.58	3.7 ± 0.61	0.121	3.64 ± 0.62	0.07
	Median [IQR (25–75)]	4 (3.52–4.27)	4 (3.38–4.01)		4 (3.35–4.0125)	
*Physical activity*	Mean ± SD	4.49 ± 0.87	4.09 ± 1.05	0.104	4.09 ± 1.05	0.10
	Median [IQR (25–75)]	4 (4‐5)	4 (3.33‐4.75)		4 (3.33‐4.75)	
*Total (CIS‐T)*	Mean ± SD	86.65 ± 13.97	78.59 ± 15.74	0.043	75.21 ± 14.6	0.005
	Median [IQR (25–75)]	84 (75.2–93.45)	77 (67.2–87.85)		75 (64.55–85.2)	
Sleep Hygiene Index Total	Median [IQR (25–75)]	25.5 (22–30)	23 (20.75–26.25)	0.141	23 (20.75–26.25)	0.11

Pearson Chi‐Square Test *p* < 0.05.

Wilcoxon test *p* < 0.05.

The risk of OSAS was low in 11 patients (36.7%) and high in 19 patients (63.3%). In the control group, 9 patients (28.1%) were found to be at low risk and 23 patients (71.9%) were found to be at high risk in the risk assessment of OSAS by applying the Berlin Form.

There was no significant difference between the MOS Sleep Scale scores of the control group patients and the intervention group patients in terms of sleep disturbance, sleep adequacy, shortness of breath, sleepiness, snoring, and sleep duration sub‐dimensions.

MOS Sleep Scale Sleep disturbance subscale scores were significantly higher in patients in the control group than in patients in the intervention group at 1 month (*p* = 0.029).

When the sleep adequacy sub‐dimension scores of the group were examined, the scores of the intervention group patients who received training in the 1st month after the training were found to be significantly lower than those of the control group patients (*p* = 0.044).

The scores of the intervention group patients who received training in the 1st month were higher than those of the control group (*p* = 0.004).

MOS Sleep Scale scores were administered to the patients in the intervention group at 2 months and compared with the control group. Although there was a significant difference between sleep disturbance, sleep adequacy, shortness of breath, and sleepiness subscale scores in the two groups, there was no statistically significant difference between snoring and sleep duration.

MOS Sleep Scale Sleep disturbance subscale scores were higher in the control group compared to the intervention group at 2 months (*p* = 0.005).

Sleep‐disordered breathing and sleepiness subscale scores were higher in the intervention group patients (*p* = 0.003) compared to the control group (*p* = 0.007) at the 2‐month assessment.

The total scores of the Fatigue Scale were lower in the intervention group patients compared to the control group after 1 month (*p* = 0.043).

Fatigue and concentration subscale scores of the group decreased significantly at the end of the 2nd month (*p* = 0.003), (*p* = 0.003).

Although the Fatigue Scale motivation and physical activity subscale scores of the patients at the end of the 2nd month were lower compared to the control group, it was not statistically significant (*p* < 0.05).

At the end of the 2nd month, the total Fatigue Scale scores of the sample were lower in the intervention group compared to the control group (*p* = 0.005).

### 3.2 Comparison of Scale Scores After the First and Second Months of the Training

The comparison of the scale scores of the intervention group patients at the end of the 1st and 2nd months is given in Table 3.

**Table 3:** Comparison of scale scores after the first and second training given to the intervention group.

		1st month after training	2nd month after training	*p*
MOS sleep scale subscales				
*UPI1*	Mean ± SD	3.87 ± 0.54	3.92 ± 0.55	0.38
	Median [IQR (25–75)]	4 (3.5–4.208)	4 (3.5–4.3)	
*UPI2*	Mean ± SD	3.68 ± 0.45	3.72 ± 0.43	0.38
	Median [IQR (25–75)]	4 (3.333–4)	4 (3.305–4)	
*Sleep Disorder*	Mean ± SD	57.17 ± 10.4	59.03 ± 9.81	0.004
	Median [IQR (25–75)]	58 (49–64)	60 (53–67)	
*Sleep Adequacy*	Mean ± SD	53.57 ± 15.9	49.17 ± 13.96	0.001
	Median [IQR (25–75)]	58 (42–67)	50 (39.75–58)	
*Shortness of breath*	Mean ± SD	83.33 ± 17.46	83.9 ± 17.73	0.32
	Median [IQR (25–75)]	83 (67–100)	83 (67–100)	
*Sleep State*	Mean ± SD	74.8 ± 14.42	77.4 ± 13.56	0.003
	Median [IQR (25–75)]	78 (61–84.5)	78 (67–89)	
*Snoring*	Mean ± SD	38.8 ± 22.86	38.8 ± 22.86	1.00
	Median [IQR (25–75)]	33 (29–37.25)	33 (29–37.25)	
*Sleep Duration*	Mean ± SD	5.00 ± 1.05	5.03 ± 1.06	0.046
	Median [IQR (25–75)]	5 (4–6)	5 (4–6)	
Fatigue Scale Subscales				
*Fatigue*	Mean ± SD	4.01 ± 1.02	3.75 ± 0.9	0.000
	Median [IQR (25–75)]	4 (3.125–4.75)	4 (3.09375–4.25)	
*Concentration*	Mean ± SD	3.89 ± 0.8	3.67 ± 0.75	0.001
	Median [IQR (25–75)]	4 (3.35–4.25)	4 (3.2–4.2)	
*Motivation*	Mean ± SD	3.7 ± 0.61	3.64 ± 0.62	0.001
	Median [IQR (25–75)]	4 (3.38–4.01)	4 (3.35–4.01)	
*Physical activity*	Mean ± SD	4.09 ± 1.05	4.09 ± 1.05	1.00
	Median [IQR (25–75)]	4 (3.33–4.75)	4 (3.3–4.75)	
*Total (CIS‐T)*	Mean ± SD	78.59 ± 15.74	75.21 ± 14.6	0.000
	Median [IQR (25–75)]	77 (67.2–87.85)	75 (64.55–85.2)	
Sleep Hygiene Index Total	Mean ± SD	24.33 ± 4.99	24.03 ± 4.69	0.041
	Median [IQR (25–75)]	23 (20.75–26.25)	23 (20.75–26.25)	

Pearson Chi‐Square Test *p* < 0.05. Wilcoxon test. *p* < 0.05.

After the sleep hygiene training was given face‐to‐face with a structured training program, data were collected in the 1st and 2nd months. Accordingly, statistically significant differences were found between MOS Sleep Scale scores. After the training, no statistically significant difference was found between the MOS Sleep Scale UPI1 (Sleep problem index1), UPI2, Shortness of breath, and Snoring sub‐dimension scores of the patients in the intervention group.

Although the patients in the intervention group received training, their sleep disturbance subscale scores were higher at the end of the 2nd month compared to the 1st month (*p* = 0.004).

Sleep adequacy subscale scores of the intervention group were significantly lower at the end of the 2nd month (*p* = 0.001).

Sleepiness subscale scores of the patients who received training were higher at the end of the 2nd month compared to the scores after the 1st month (*p* = 0.003).

According to the data of the patients at the end of the 2nd month, quantitative sleep duration increased slightly (*p* = 0.046).

Concentration and motivation subscale scores of the patients were lower at the end of the 2nd month compared to the 1st month (*p* = 0.001), (*p* = 0.001). Fatigue Scale total scores of the patients in the intervention group who received sleep hygiene education were found to be lower at the end of the 2nd month compared to the scores at the end of the 1st month (*p* = 0.000). A statistically significant, albeit borderline, decrease was found between the Sleep Hygiene Index scores of the patients in the intervention group after 1 and 2 months (*p* = 0.041).

### 3.3 Comparison of the Scores of the MOS Sleep Scale, Berlin Form, Fatigue Scale, and Sleep Hygiene Index Scales

Although not given as a table, when the correlation of scale scores with each other is examined, A statistically significant negative and weak to moderate correlation was found between the MOS Sleep Scale and Berlin Form scores, and Fatigue Scale scores (*p* < 0.05). A statistically significant positive, moderate correlation was found between the Berlin Form and Fatigue Scale scores (*r* = 0.389; *p* < 0.05), and the Fatigue Scale and Sleep Hygiene Index scores (*r* = 0.475; *p* < 0.05).

### 3.4 Comparison of Scale Scores and Symptoms

The scale scores and amount of coverage are given in Table 4.

**Table 4:** Comparison of mos sleep scale, berlin form, fatigue scale and sleep hygiene index scores in terms of the presence of symptoms experienced by the patients.

		Mos sleep scale		Berlin form		Fatigue scale CIS‐T		Sleep hygiene index	
		YES	NO	YES	NO	YES	NO	YES	NO
Respiratory arrest	Mean ± SD	42.02 ± 6.13	44.92 ± 5.42	6.89 ± 1.40	4 ± 1.55	86.20 ± 16.51	77.63 ± 11.75	25 ± 5.88	26.28 ± 5.57
	Median (IQR (25–75)	44 (8)	46 (8)	7 (2)	4 (2)	84.2 (18.4)	79 (12.8)	23 (9)	25 (6)
	P	0.063		< 0.001		0.075		0.306	
Sudden awakening	Mean ± SD	42.58 ± 6.24	44.38 ± 5.41	6.48 ± 1.64	4.23 ± 1.94	85.48 ± 15.50	77.40 ± 13.62	26 ± 6.30	24.57 ± 4.47
	Median [IQR (25–75)]	44 (9)	43 (8)	7 (3)	4 (2)	83 (16.8)	83 (21.8)	25 (8)	23 (4)
	P	0.300		< 0.001		0.185		0.502	
Dry mouth	Mean ± SD	42.8 ± 5.97	44.83 ± 6.03	5.94 ± 1.84	4.83 ± 2.62	84.92 ± 15.29	73.68 ± 11.89	26.08 ± 5.63	23.16 ± 5.89
	Median [IQR (25–75)]	43 (8)	47 (6.5)	6 (4)	4.5 (4.5)	84.1 (16.8)	71.4 (8.2)	25 (8)	23 (8.5)
	P	0.202		0.132		0.020		0.091	
Insomnia	Mean ± SD	38.54 ± 5.31	45.75 ± 4.69	6.95 ± 1.25	5.05 ± 2.08	94.59 ± 14.27	76.23 ± 11.51	28.72 ± 6.46	23.75 ± 4.49
	Median [IQR (25–75)]	38(8)	46 (6.5)	7 (2)	5 (4)	90.2 (25.4)	75.3 (15.3)	27 (11)	23 (6)
	P	< 0.001		< 0.001		< 0.001		0.003	
Morning headache	Mean ± SD	40.90 ± 5.61	45.63 ± 5.46	6.37 ± 1.71	5.03 ± 2.15	88.93 ± 16.00	76.14 ± 11.39	27.46 ± 6.03	23.43 ± 4.68
	Median [IQR (25–75)]	41.5 (8.5)	47 (7)	7 (3)	4.5 (4)	87.2 (16.4)	75.8 (15.2)	26.5 (9.5)	23 (6)
	P	0.002		0.029		0.002		0.008	
Depressive state	Mean ± SD	36.87 ± 4.88	44.12 ± 5.58	7.5 ± 1.06	5.46 ± 2.02	103.6 ± 13.73	79.64 ± 12.95	28.25 ± 5.06	25.11 ± 5.77
	Median [IQR (25–75)]	37 (7.5)	45 (8)	8 (1.5)	5 (3)	102.6 (27.5)	79.5 (15.6)	28.5 (9)	23 (6)
	P	0.003		0.017		< 0.001		0.107	
Irritability	Mean ± SD	40.73 ± 5.61	44.97 ± 5.67	7.19 ± 1.23	4.66 ± 1.85	85.5 ± 16.50	80.76 ± 14.23	24.96 ± 6.07	25.91 ± 5.55
	Median [IQR (25–75)]	41.5 (9)	45.5 (8)	7 (1)	4 (3)	85.3 (17.2)	80.2 (14.5)	24 (10)	24 (6.5)
	P	0.006		< 0.001		0.325		0.402	
Attention problem	Mean ± SD	38.76 ± 5.63	44.36 ± 5.56	6.92 ± 1.38	5.40 ± 2.08	93.03 ± 14.20	80.02 ± 14.48	29.23 ± 5.84	24.53 ± 5.36
	Median [IQR (25–75)]	39 (8)	45 (8)	7 (2)	5 (3)	90.2 (9.4)	79 (15.6)	27 (9)	23 (6)
	P	0.004		0.024		0.005		0.008	

Mann Whitney‐U Test *p* < 0.05.

A statistically significant difference was found between Berlin Form scores according to the presence of respiratory arrest and sudden awakening complaints (*p* < 0.001). Accordingly, it was determined that the Berlin Form scores of individuals with respiratory arrest and sudden awakening complaints were higher than those without these complaints.

There was a statistically significant difference between the MOS Sleep Scale, Berlin Form, Fatigue Scale, and Sleep Hygiene Index scores according to the presence of insomnia, morning headache, depressive state, and attention problems (*p* < 0.05). The Berlin Form, Fatigue Scale, and Sleep Hygiene Index scores of patients with complaints of insomnia, morning headache, depressive state, and attention problems were higher than those of patients without these complaints, while the MOS Sleep Scale scores were lower than those of patients without insomnia, morning headache, depressive state, and attention problems.

## 4 Discussion

This study emphasizes the importance of the effect of sleep hygiene education on OSAS symptoms, sleep quality, and fatigue in patients with AF. A sleep hygiene education was not effective in the risk of developing OSAS. Typical symptoms such as snoring and daytime sleepiness are not reliable markers of sleep apnea, especially in patients with AF, and even questionnaire‐type screening tools have limited predictive value in the evaluation of OSAS in patients with AF [19].

The significant difference between the sleep disturbance, sleep adequacy, shortness of breath, and sleepiness sub‐dimension scores indicates that the sleep hygiene training given is effective in terms of these sub‐dimensions, while it is not effective on snoring and sleep duration [20]. Similar to our study findings, Wong et al. (2017) reported a decrease in the severity of insomnia in individuals with behavioral therapy programs including sleep hygiene practices and relaxation training [21]. there are studies suggesting that cognitive behavioral therapy including sleep hygiene training has positive effects on sleep quality and OSAS symptoms [22, 23].

Fatigue Scale fatigue, concentration, motivation, and physical activity sub‐dimension scores of the patients in the intervention group were lower than the control group patients according to the evaluation made at the end of the 1st month, it was not statistically significant. The fact that the fatigue and concentration sub‐dimension scores of the intervention group decreased significantly at the end of the 2nd month suggests that the sleep hygiene training given was not directly effective on the fatigue symptom.

Low MOS Sleep Scale scores may indicate better sleep quality and a lower risk of sleep apnea, while high scores may indicate poorer sleep quality and a higher risk of sleep apnea. Thus, low MOS Sleep Scale scores are generally associated with less fatigue, while high scores may be associated with more fatigue. However, individual differences and other factors may also influence this association.

In our study, a statistically significant negative and weak correlation was found between the MOS Sleep Scale and Sleep Hygiene Index scores, indicating that sleep quality may be affected by sleep habits and environmental factors. However, a patient with a good Sleep Hygiene Index may still have poor sleep quality or vice versa. Zhu et al. (2023) (N = 315 patients) reported that although individuals had high Sleep Hygiene Index scores, conditions such as anxiety, depression, hopelessness, and lack of social support negatively affected sleep quality [7].

Berlin Form scores of individuals with respiratory arrest and sudden awakening complaints were higher than those without these complaints. Accordingly, it can be concluded that the risk of developing OSAS is higher in individuals with respiratory arrest and sudden awakening complaints. The Berlin Form, Fatigue Scale and Sleep Hygiene Index scores of patients with insomnia, morning headache, depressive mood, and attention problems were higher than those without these complaints, while MOS Sleep Scale scores were lower than those without insomnia, morning headache, depressive mood and attention problems. Complaints such as sleep disturbance, shortness of breath, waking up with a headache, and sleepiness during the day negatively affect sleep quality and increase the risk of developing OSAS [24, 25, 26].

## 5 Conclusion

Although structured sleep hygiene education given to patients diagnosed with AF positively affects sleep duration, it is ineffective in ensuring that patients have quality sleep in which they get enough rest and do not experience fatigue. The effect of the training decreases as time passes. When sleep quality is not ensured, sleep apnea increases. Symptoms in patients (insomnia, morning headache, depression, attention problems) negatively affects sleep quality.

Sleep hygiene practices are easy, inexpensive, and improve sleep quality, they are not sufficient alone. It is recommended that planned sleep hygiene programs should be supported by digital health applications, cognitive behavioral therapies, and medical interventions, especially in patients diagnosed with AF with symptoms of OSAS.

## Author Contributions

**Besey Ören:** Study conception, designed data analysis, approved the final version to be published. **Semiha Akin Eroglu:** Study conception, designed data analysis, approved the final version to be published. **Busra Zehra Buyukkilic:** Study conception, designed data analysis, wrote the first draft of the manuscript, and approved the final version to be published. **Ahmet Lutfullah Orhan:** Wrote the first draft of the manuscript, and approved the final version to be published.

## Ethics Statement

Written approval (decision number: 11/18, dated: 25/05/2023) was received from the Scientific Research Ethics Committee of Health Sciences University to implement the study. Institutional permission was obtained for data collection.

## Consent

All participants were informed about the research and their informed consent was received.

## Conflicts of Interest

The authors declare no conflicts of interest.

## Supporting information

CONSORT‐Checklist.doc.

## Data Availability

The data obtained in this study can be shared upon request for use by the authors.
